# Using citizen science data to monitor the Sustainable Development Goals: a bottom-up analysis

**DOI:** 10.1007/s11625-021-01001-1

**Published:** 2021-07-23

**Authors:** Laura Ballerini, Sylvia I. Bergh

**Affiliations:** 1 Vrije Universiteit, Amsterdam, The Netherlands; 2 University of Oxford, Oxford, UK; 3 The Hague University of Applied Sciences, The Hague, The Netherlands; 4 International Institute of Social Studies, Erasmus University Rotterdam, Rotterdam, The Netherlands

**Keywords:** Sustainable Development Goals, United Nations, Citizen science data, Citizen science, Monitoring SDG indicators

## Abstract

Official data are not sufficient for monitoring the United Nations Sustainable Development Goals (SDGs): they do not reach remote locations or marginalized populations and can be manipulated by governments. Citizen science data (CSD), defined as data that citizens voluntarily gather by employing a wide range of technologies and methodologies, could help to tackle these problems and ultimately improve SDG monitoring. However, the link between CSD and the SDGs is still understudied. This article aims to develop an empirical understanding of the CSD-SDG link by focusing on the perspective of projects which employ CSD. Specifically, the article presents primary and secondary qualitative data collected on 30 of these projects and an explorative comparative case study analysis. It finds that projects which use CSD recognize that the SDGs can provide a valuable framework and legitimacy, as well as attract funding, visibility, and partnerships. But, at the same time, the article reveals that these projects also encounter several barriers with respect to the SDGs: a widespread lack of knowledge of the goals, combined with frustration and political resistance towards the UN, may deter these projects from contributing their data to the SDG monitoring apparatus.

**Supplementary Information:**

The online version contains supplementary material available at 10.1007/s11625-021-01001-1.

## Introduction

In 2015, the United Nations (UN) launched its most ambitious development plan: the Sustainable Development Goals (SDGs), a set of 17 goals, 169 targets, and 232 indicators intended to “end all forms of poverty, fight inequalities and tackle climate change” by 2030, worldwide (Lämmerhirt et al. 2017, p. 8; for definitions and an example of the SDGs, see Table 1).

**Table 1:** Definitions and example of the SDG goals, targets, and indicators

	Goal	Target	Indicator
Key definition	An ambitious commitment to an outcome addressing a single challenge	A specific, measurable and time-bound action directly contributing to the goals	An output metric used to measure progress towards the target
Example	4. Ensure inclusive and equitable quality education and promote lifelong learning opportunities for all	4.2 By 2030, ensure that all girls and boys have access to quality early childhood development, care and pre-primary education so that they are ready for primary education	4.2.1 Proportion of children under 5 years of age who are developmentally on track in health, learning and psychosocial well-being, by sex

Sources: Lämmerhirt et al. (2017), UNGA (2015)

From a statistical perspective, monitoring countries’ progress towards these goals has been described as an “unprecedented statistical challenge” (MacFeely 2018, p. 5). Not only do the SDGs require a staggering amount of data, but they also need data that are high in quality, broad in coverage, frequently available, and spatially disaggregated (Fritz et al. 2019). At the moment, SDG monitoring mostly relies on traditional and official data, which are primarily collected by national statistical offices (NSOs) (Daguitan et al. 2019a, b).^1^ Albeit necessary and valuable, official data have, however, several shortcomings (Fisher and Fukuda-Parr 2019; Satterthwaite and Dhital 2019).

Firstly, there are technical limitations: official data require costly and lengthy collection processes, and they have limited spatial variation and coverage (MacFeely 2018). This results in infrequent data collection cycles, huge data gaps,^2^ and unrepresentative samples, from which marginalized populations, such as indigenous people (Yap and Watene 2019), and delicate issues, such as women’s sexual and reproductive health rights (Yamin 2019), are systematically excluded. Moreover, the widespread use of official data within the SDG framework raises political concerns. Indeed, scholars find that public officials frequently abuse their firm grip on these data to hide contentious information, gloss over their countries’ problems, or artificially boost their performances with respect to the SDGs (Fukuda-Parr and McNeill 2019; Langford and Winkler 2014). Lastly, official data are often epistemically ill-suited to capture contextual information and local knowledge (Yamin 2019). With their aura of objectivity and neutrality, these metrics tend to conceal precious qualitative details, turning local politics and ideologies into abstract statistics and averages (Merry 2019).

Against the backdrop of these shortcomings, a burgeoning scholarship is proposing to complement the official statistics used for SDG reporting with unofficial and alternative sources of data (MacFeely and Nastav 2019; Saner et al. 2019; Winkler and Satterthwaite 2017). One such source is ‘citizen science data’ (CSD).^3^ CSD consist of data that ‘citizen scientists’ voluntarily generate and gather by employing a wide range of technologies and participatory collection methodologies, such as community-based monitoring, crowdsourcing online platforms, or digital sensors (DataShift 2017; Gray et al. 2016). In most cases, the production of CSD is initiated by citizens or civil society organizations and is supervised by a variegated set of intermediary actors, among which non-governmental organizations (NGOs), academic researchers, private companies, and government agencies. The voluntary and participatory nature of CSD marks their distinguishing traits and set them apart from other types of data such as ‘big data’ (Mahajan 2019; Meijer and Potjer 2018).^4^

CSD are usually gathered in the context of ‘citizen science projects’ to which we refer to as ‘CS projects’ or ‘CS initiatives’ (Cohn 2008). As defined by Eitzel et al. (2017), ‘citizen science’ is a rather novel scientific field which engages members of the public in the process of creating scientific knowledge and addressing real-world problems (see also Bonney et al. 2009, 2016; Cohn 2008; Haklay, 2020; Jordan et al. 2015; Kosmala et al. 2016).^5^ It revolves around the idea of citizens as “amateur scientists” (Lukyanenko et al. 2011, p. 465) or “fieldworkers of their own lives” (Purdam 2014, p. 375), who partner with professional scientists to gather data on a panoply of different subjects (Wiggins and Crowston 2011). Among several other fields, CSD have been collected for projects related to biology (Sullivan et al. 2009), ecology (Shirk et al. 2012), environmental sciences (Silverton 2009), biodiversity (Burgess et al. 2017), hydrology (Lowry and Fienen 2013) and sociology (Purdam 2014). Concrete examples of CSD include bird observations,^6^ records of violations of indigenous rights,^7^ and interactive maps of the spread of COVID-19.^8^

Both theoretically and empirically, researchers pinpoint the benefits and drawbacks of CSD (Blaney et al. 2016). The most intuitive benefits of these data include their fine-grained resolution, and time- and cost-effectiveness (Burgess et al. 2017; Carlson and Cohen 2018). Related advantages are that CSD can reach remote locations, foreground the concerns of marginalized populations, and raise public participation (Fritz et al. 2019; Gabrys et al. 2016; West and Pateman 2017). Drawbacks of CSD comprise their potentially low interoperability, limited sustainability, urban bias (i.e. the fact that CSD are mostly collected in cities), and contested data quality and reliability (Bishop et al. 2020; Wiggins and Crowston 2011). These problems are frequently the result of a lack of funding, citizens’ fluctuating levels of engagement, and the reliance on non-standardized data collection methodologies (Danielsen et al. 2005; Freitag et al. 2016). Recent studies, however, show that new technologies and innovative approaches are contributing to successfully tackling some of these issues and to tremendously augmenting the quality and reach of CSD (Bishop et al. 2020; Burgess et al. 2017; Gabrys et al. 2016; Kosmala et al. 2016).

These promising advancements in citizen science spurred a handful of scholars to take CSD into the field of global development (Flückiger and Seth 2016; Fraisl et al. 2020), and to assess the possibilities for CSD to be used to monitor the SDGs. Following the publication of some non-academic reports (DataShift 2017; Lämmerhirt et al. 2017, 2018; West and Pateman 2017), the first academic article entirely dedicated to this issue was published by Fritz et al. in October 2019. It argues that CSD could fill key spatial and temporal SDG data gaps, and help to engage the general public. In this way, CSD could also foster progress towards the achievement of several targets related to inclusion and participation. In a follow-up article published in this journal (i.e. Sustainability Science), Fraisl et al. (2020) empirically test their argument by analysing the current data gaps in SDG indicators and identifying CS projects that could help to fill these gaps. Their findings show that CS projects could provide data on over 30 percent of the SDG indicators, although only a few are currently doing so. The reasons for why CSD are so rarely used within the SDG framework can be found in technical and statistical impediments, but also in the existence of widespread skepticism towards citizen science, coupled with governments’ fear of losing control over the data (see also Lukyanenko et al. 2016; MacFeely 2018; Winkler and Satterthwaite 2017).

These recent studies and results are opening the door to a new strand of literature exclusively devoted to studying the link between citizen science and the SDGs. Yet, this literature is still in its infancy and more academic inquiries are urgently warranted (personal communication with Dilek Fraisl, International Institute for Applied Systems Analysis, 2020; Schade et al. 2020). One particular limitation of existing research is that it is investigating the relationship between CSD and the SDGs primarily from the perspective of the SDGs (Fraisl et al. 2020; Lämmerhirt et al. 2018). This means that researchers have so far mainly relied on top-down research (from the SDGs to CSD), examining how SDG monitoring can benefit from CSD, but essentially assuming that CS initiatives are able and willing to contribute to the SDGs.

In short, scholars have so far not asked how CS projects view and experience their engagements with the SDGs or, more fundamentally, whether these projects even can and want to link their data to the SDGs (Bett et al. 2020). In this way, the literature has partly overlooked the perspective of CS projects and given a one-sided portrayal of the link between CSD and the SDGs. To the best of our knowledge, no attempts have been made yet to take a bottom-up approach and analyse the CSD-SDG relationship from the perspective of CS projects. Without taking such an approach, however, our understanding of the CSD–SDG link remains limited, and crucial questions are left unexplored.

This article aims to bridge this gap in the literature. Taking a bottom-up rather than a top-down approach, this research gives voice to CS projects, with the ultimate goal of creating a more balanced and complete understanding of the CSD-SDG link. Specifically, this article focuses on three research questions (RQs):

1. For which purposes do CS projects produce data?
2. What types of involvements do CS projects have with the SDGs?
3. What do CS projects see as the main opportunities and barriers in linking CSD to the SDGs?

To explore these questions, the remainder of this article is structured as follows. The next section briefly reviews the available literature on our three research questions. In Sect. 3, the methodology and limitations of the study are discussed. Section 4 presents the results and discussion, while Sect. 5 concludes and points to some key implications for policy and future research.

## The link between CSD and the SDGs

Although there is a conspicuous paucity of empirical research addressing the first research question (on the purposes for which CS projects produce data), the citizen science literature provides some theoretical grounding. It identifies three core purposes of CSD: community, public governance, and scientific purposes (Bio Innovation Service 2018; Kieslinger et al. 2017; Turrini et al. 2018). The community purpose refers to the fact that the majority of CS projects produce data to generate local impacts and individual gains, which materialize in positive learning effects for participants (in terms, for instance, of improved skills and deeper scientific knowledge) as well as higher levels of engagement and empowerment (see Wiggins and Crowston 2011 on ‘Action’ and ‘Education’ CS projects). Moving beyond the local domain, Meijer and Potjer (2018) show that CSD can influence public governance by (1) increasing collaboration between citizens and public officials, (2) enhancing government accountability and transparency, and (3) raising public officials’ awareness of certain issues (see also Ponti and Craglia 2020). Lastly, CSD are often produced to inform scientific research and advance scientific progress (Newman et al. 2017; Turbé et al. 2019).

On the second question (what types of involvements do CS projects have with the SDGs?), a thorough literature search yielded no academic study and only scarce grey literature that attempts to directly answer this question (West and Pateman 2017). Within the context of their research for the thinktank DataShift, Lämmerhirt et al. (2017, p. 15) analyse a variety of CS projects and find that these initiatives rarely engage with the SDGs, “beyond perhaps requesting funding from high-level donors” who are involved with the UN goals. Other researchers confirm these findings by showing that only a few initiatives mention the SDGs on their websites (Schade et al. 2020). However, they do not empirically check whether website mentions can be used to determine the level of engagement of CS projects with the SDGs.

The third question brings into focus the opinions held by CS projects on the SDGs by asking: What do CS projects see as the main opportunities and barriers in linking CSD to the SDGs? As with the second question, non-academic reports are the only available sources addressing this issue—and do so only partially. Specifically, these reports show that some CSD initiatives favourably welcome the integration of the SDGs since these goals can “provide a common language to move towards integrated development” (Lämmerhirt et al. 2017, p. 27). In other words, the international standing of the SDG framework can legitimize and guide the work of CS projects and motivate their communities. Moreover, CS initiatives can receive concrete support through the SDGs by using these goals to attract financial investments and foster new partnerships (Lämmerhirt et al. 2018, p. 58).

However, these projects also face some obstacles. They may refrain from linking their work to the SDGs due to a lack of knowledge of the goals and their indicators (Lämmerhirt et al. 2017, p. 15). Even when they are aware of the SDGs, some CS projects see insurmountable technical challenges posed by the UN goals. In fact, the SDG indicators are built on very specific methodologies, proxies, and criteria, which can be difficult to replicate in the context of CS projects often relying on qualitative data and non-standardized methods (Schade et al. 2020; See et al. 2020).

In sum, a careful review of the academic and grey literature shows that the link between CSD and the SDGs is still understudied and that there are currently only a few tentative answers to our three research questions, most of which have not been empirically tested (for a summary of the literature’s answers, see Table 2).

**Table 2:** Overview of the answers to the three research questions found in the literature

Research question	Literature answers	References
**CSD purposes**		
For which purposes do CS projects produce data?	Communities	Kieslinger et al. (2017), Turrini et al. (2018)
	Public governance	Meijer and Potjer (2018), Ponti and Craglia (2020)
	Scientific research	Newman et al. (2017), Turbé et al. (2019)
**SDG involvements**		
What types of involvements do CS projects have with the SDGs?	Grant applications Website mention	Lämmerhirt et al. (2017) Schade et al. (2020)
**SDG opportunities and barriers**		
What do CS projects see as the main opportunities and barriers in linking CSD to the SDGs?	**Opportunities**	
	Framework	Lämmerhirt et al. (2017)
	Concrete support	Lämmerhirt et al. (2018)
	**Barriers**	
	Lack of knowledge	Lämmerhirt et al. (2017)
	Technical	Schade et al. (2020), See et al. (2020)

## Research methodology

### Research design

To empirically investigate the three research questions, this study took a bottom-up approach and examined the perspectives of CS projects through an analysis of 30 case studies. To this end, we employed an explorative comparative case study research design (Yin 2014). The choice of this research design is particularly appropriate for shedding light on the link between CSD and the SDGs given the conspicuous paucity of research on the matter and the ample opportunity to learn by simultaneously examining different cases (Turbé et al. 2019).

With regard to the sampling procedure, we identified case studies through a three-step search strategy, given the absence of a database that includes a broad topical range of CS projects.^9^ Firstly, the first author performed an iterative Google search of CS projects using keywords widely found in the literature such as “citizen science data”, “citizen-generated data”, and “community-generated data” (Rogers 2013).^10^ Secondly, the first author contacted key researchers in the field (Fraisl 2020; personal communication with Jonathan Gray, King’s College London, 2020; personal communication with Albert Meijer, Utrecht University, 2020) and followed four webinars that directed us towards more CS initiatives (see Bett et al. 2020; Schade et al. 2020; See et al. 2020; Ward et al. 2020). Lastly, we applied a so-called snowballing sampling technique, using the cases that we had already identified to find more initiatives (Biernacki and Waldorf 1981).

The combination of these search strategies helped us to compile an inventory of 231 CS projects (see Table S1 in the Supplementary Material). Apart from the name, website, and a brief description, each project in the inventory is characterized by the following attributes^11^: organization category, geographic coverage, sector(s), and main technologies. Although extensive, it should be noted that this inventory does not pretend to provide an exhaustive list of all CS projects around the world; rather, its illustrative set of case studies is intended as a starting point from which to explore the breadth and depth of the field of citizen science and its link to the SDGs.

After identifying the 231 CS projects and gathering key information on them, we took an approach analogous to Meijer and Potjer (2018) and applied ‘selective’ and ‘diversifying’ criteria to restrict our sample (see also Eisenhardt 1989). In particular, cases were chosen according to the selective criteria that they:

1. Involve the production and use of CSD, as defined above (i.e. data that citizens voluntarily generate and gather by employing a wide range of participatory collection methodologies and technologies). This selective criterion ensured that projects that inappropriately use the term CSD were omitted.^12^
2. Are currently implemented. Past CS projects were excluded to avoid the risk of gathering outdated information and relying on non-functioning websites.
3. Provide enough information for the analysis. CS projects that have little material available online, are not linked to any relevant publication, and whose representatives could not be reached were not included in the final sample.

On top of these selective criteria, we also sought diversity in the final sample by taking into consideration the following criteria: (1) sector(s), (2) geographic coverage, (3) organization category, (4) size, and (5) technologies. The decision to map for diversity, alongside relevance, was motivated by the idea that the final sample of cases should reflect the versatile nature of CSD and their widespread utilizations in developed, transitioning, and developing countries. Moreover, we opted for a diverse sample to show that CSD are adaptable and applicable to a wide range of SDGs. By combining selective and diversifying criteria, we settled on a final sample of 30 CS initiatives (see Table 3).

**Table 3:** Overview of the 30 CS projects

CS project	Website	Description	Sector(s)	Category	Technology	Coverage
Africa’s Voices	https://www.africasvoices.org	Social enterprise that gathers opinions and concerns of African citizens on issues such as public health and gender equality	Health, gender	Non-governmental	Interactive radio and SMS surveys	Africa
CoCoRaHS	https://www.cocorahs.org	Network of volunteers that uses low-cost monitoring tools to measure and map precipitation across North America	Meteorology	Non-governmental	Monitoring kit	Canada and USA
DETECT	https://detectstudy.org	Citizen scientists share their wearable data and self-report symptoms and lab results to improve the identification of viral illnesses	Health	Non-governmental	Wearable devices	USA
Did you feel it?	https://earthquake.usgs.gov/data/dyfi/	Initiative that collects information from people who experienced an earthquake and creates maps on the extent of the damage	Natural hazards	Governmental	Surveys	Global
EUPATI	https://www.eupati.eu	Patient communities contribute to medical research by sharing their experiences with illnesses and medical treatments	Health	Non-governmental	Surveys	Europe
European Alien Species Information Network (EASIN)	https://www.ffm.de/frankfurt/de/home	Citizens collect data on alien species to monitor biological invasions and support EU regulations on invasive alien species	Biodiversity	Governmental	App	Europe
FixMyStreet	https://www.fixmystreet.com	Independent website that gathers citizen reports on the state of the streets in their communities and sends them to local councils	Public space	Non-governmental	App	UK
FreshWater Watch	https://freshwaterwatch.thewaterhub.org	Project that asks citizen scientists to monitor the water quality of their local streams	Water quality	Non-governmental	Monitoring kit	Global
Hush City	http://www.opensourcesoundscapes.org	Citizen science mobile app through which people identify and assess quiet areas	Quiet areas	Academic	App	Global
I Like Clean Air	http://www.ilikecleanair.org.uk	Initiative that helps parents and children to monitor air pollution around London schools	Air quality	Community-led	Monitoring kit	London
iNaturalist	https://www.inaturalist.org	Organization that gathers people’s observations of plants and animals, helping them through the identification process	Biodiversity	Non-governmental	App	Global
InfoAmazonia	https://infoamazonia.org	News agency that gathers citizen reports on the endangered areas of the Amazon region	Forest conservation	Non-governmental	Platform	South America
LANDex	https://www.landexglobal.org/en/	Platform that collects people-based assessments of land-related issues and measures land data using people-centred indicators	Land usage	Consortium	Platform	Global
Land Matrix	https://landmatrix.org	Online open-access platform that captures and shares citizen-generated data about large-scale land acquisitions	Land usage	Consortium	Platform	Global
Map Kibera	https://mapkibera.org	Project that helps young residents of Kenyan slums to create digital maps of their communities	Poverty, education	Non-governmental	Interactive map	Kenya
Marine Debris Tracker	https://marinedebris.engr.uga.edu	Open-data tool used by citizen scientists to record data on inland and marine debris	Debris	Academic	App	Global
Mosquito Alert	http://www.mosquitoalert.com/en/	Cooperative citizen science observatory that collects observations of the tiger mosquito and the yellow fever mosquito	Health	Consortium	App	Global
Nepal Monitor	https://www.nepalmonitor.org	Map that collects and locates any sort of violence-related incidents and provides reports to concerned stakeholders	Human rights	Non-governmental	Surveys and interactive map	Nepal
Open Humans	https://www.openhumans.org	Organization that empowers individuals and communities around their personal health data	Citizen participation	Non-governmental	Platform	Global
OpenLitterMap	https://openlittermap.com	Online map that provides crowdsourced information on problems related to plastic pollution	Debris	Community-led	Interactive map	Global
QuestaGame	https://questagame.com	Outdoor multiplier online game in which individuals take pictures of rare plants and animals and compete to identify them	Biodiversity	Private sector	Online game	Global
Safecast	https://safecast.org	Platform, launched in the wake of the Fukushima disaster, through which volunteers collect radiation measurements and air-quality data	Air quality	Community-led	Platform	Global
SciStarter	https://scistarter.org	Search engine that connects CS organizations to citizen scientists	Citizen participation	Academic	Platform	Global
SeeClickFix	https://seeclickfix.com	App that gathers complaints of street problems and sends the data to city councils	Public space	Private sector	App	Canada and USA
Stall Catchers	https://stallcatchers.com	Online citizen science game through which individuals identify stalls in blood vessels and help to speed up Alzheimer’s research	Health	Non-governmental	Online game	Global
The Sentinel Project / WikiRumours	https://www.wikirumours.org	Platform on which citizens report harmful rumours and misinformation in order to prevent intercommunal conflicts	Conflict prevention	Non-governmental	App and SMS surveys	Global
USA National Phenology Network	https://www.usanpn.org/usa-national-phenology-network	Initiative that asks volunteers to record long-term observations of plant and animal life stages	Phenology	Academic	Surveys	USA
WikiMafia	https://www.wikimafia.it	Free crowdsourced encyclopaedia devoted to mapping the Mafia phenomenon in Italy	Corruption	Community-led	App	Italy
WildTrack	https://wildtrack.org	Organization that engages citizens to submit images of footprints in order to identify species and mitigate human–wildlife conflict	Wildlife conservation	Consortium	App and Cameras	Global
ZanzaMapp	https://www.zanzamapp.it	App through which citizens report the location of mosquitoes and prevent the spread of viruses such as Zika and dengue	Health	Academic	App	Italy

The final sample includes CS projects that focus on sectors which range from biodiversity and air quality to health and corruption. Thirteen of these projects are run by non-governmental organizations, while the rest are led by  governmental, academic, community, and private-sector organizations or consortia. A wide range of funding streams—such as commissioned research, government grants, and private donations—support these projects. The majority are based in the USA, Europe, or Australia, but there are also some from Africa, Asia, and South America. With respect to the geographic coverage, many operate at the global level, some have a regional focus, a few are country-specific, and one is only active in the city of London. The size of the contributing citizens varies from 250 to over 50,000.

### Data collection

Following common practices in explorative comparative case study research, the first author collected data on the 30 CS initiatives by drawing from disparate data sources: (1) projects’ websites, (2) secondary sources, and (3) personal interviews. We relied on projects’ websites to obtain information on CSD purposes (RQ 1) and check whether there was any mention of the SDGs (RQ 2). Secondary sources—comprising scientific papers, non-academic reports, official documents, newspaper articles, and podcasts—were used to complement the information collected from projects’ websites.

In addition, the first author collected primary qualitative data by directly approaching representatives of the selected initiatives via email, Facebook, LinkedIn, and web chats. Each person was given the option of either filling in an online questionnaire or participating in a semi-structured interview: 17 representatives opted to complete the online questionnaire and 13 agreed to hold an interview (see Table 4 for the complete list of respondents from CS projects).

**Table 4:** List of respondents from CS projects

No	Name	CS project	Role	Date	Platform	Code
**Respondents who filled in the online questionnaire**						
1	Bastian Greshake Tzovaras	Open Humans	Director of Research	22.05.2020	Google Form	GF01
2	Zoe Jewell	WildTrack	President	22.05.2020	Google Form	GF02
3	David Wald	Did you feel it?	Director of Research	22.05.2020	Google Form	GF03
4	Theresa Crimmins	USA National Phenology Network	Director	22.05.2020	Google Form	GF04
5	Celia L. Cañizares	European Alien Species Information Network	Consultant	25.05.2020	Google Form	GF05
6	Christopher Tuckwood	The Sentinel Project/WikiRumours	Executive Director	25.05.2020	Google Form	GF06
7	Alex Richter-Boix	Mosquito Alert	Communication Scientist	26.05.2020	Google Form	GF07
8	Tamás Bereczky	EUPATI	Courses and Content	26.05.2020	Google Form	GF08
9	Myfanwy Nixon	FixMyStreet	Marketing Manager	26.05.2020	Google Form	GF09
10	McKay Burley	SeeClickFix	Consultant	26.05.2020	Google Form	GF10
11	Sean Bonner	Safecast	Co-founder	27.05.2020	Google Form	GF11
12	Eglė M. Ramanauskaitė	Stall Catchers	Citizen Science Coordinator	01.06.2020	Google Form	GF12
13	Noah Newman	CoCoRaHS	Education and Outreach	02.06.2020	Google Form	GF13
14		Map Kibera		03.06.2020	Google Form	GF14
15	Shazia Ali	I Like Clean Air	Program Manager	04.06.2020	Google Form	GF15
16	Mahesh Bhatta	Nepal Monitor	Program Coordinator	08.06.2020	Google Form	GF16
17	Gustavo Faleiros	InfoAmazonia	Executive Director	12.06.2020	Google Form	GF17
**Respondents who took part in the interview**						
18	Caroline Nickerson	SciStarter	Program Manager	23.05.2020	Zoom	IN01
19	Pierpaolo Farina	WikiMafia	Founder	27.05.2020	Telephone	IN02
20	Cesare Bianchi	ZanzaMapp	Founder	27.05.2020	Telephone	IN03
21	Tony Iwane	iNaturalist	Community Coordinator	27.05.2020	Zoom	IN04
22	Elena Georgalla	Africa's Voices	External Relations	28.05.2020	Zoom	IN05
23	Seán Lynch	OpenLitterMap	Founder	28.05.2020	Telephone	IN06
24	Isabel Bishop	FreshWater Watch	Research Manager	01.06.2020	Zoom	IN07
25	Ward Anseeuw	LANDex	Founder	02.06.2020	Telephone	IN08
26	Ward Anseeuw	Land Matrix	Founder	02.06.2020	Telephone	IN09
27	Kathryn M. Youngblood	Marine Debris Tracker	Citizen Science Director	04.05.2020	Skype	IN10
28	Antonella Radicchi	Hush City	Founder	05.06.2020	Zoom	IN11
29	Jennifer Radin	DETECT	Epidemiologist	05.06.2020	Skype	IN12
30	Andrew Robinson	QuestaGame	Founder & Engineer	06.06.2020	Telephone	IN13

The names of those respondents who did not consent to be mentioned with their full names are suppressed here

The online questionnaire comprised three sections: the first one contained general questions about the participant and the project, the second one asked the respondent to elaborate on the purpose of CSD (RQ 1), and the last one focused on the link between the CSD and the SDGs (RQ 2 and RQ 3). The interviews were held via Zoom, Skype, and telephone and lasted between 30 and 75 min. Relevant parts were transcribed verbatim and complemented with notes taken while interviewing. The interviews followed the same format as that of the online questionnaire, with the three research questions—CSD purposes, SDG involvements, and SDG opportunities and barriers—forming their basic structure. The focus of both questionnaire and interviews lay particularly in the last two questions, given that almost no information could be found on them by only examining websites and secondary sources.

### Limitations of the research

The research methodology employed in this article has some limitations. Firstly, the search strategies used to identify and select the 30 case studies may have resulted in biases towards large-scale initiatives and those with an Internet presence. Relatedly, the keywords and criteria used to identify these case studies as well as our language skills may have led us to miss some key projects and to overrepresent English, Italian, Spanish, French, Dutch, and German initiatives. Another limitation stems from the fact that the answers given to the online questionnaire and during the interviews were often of differing quality and, thus, hard to compare: the former were short and sometimes vague, while the latter were more elaborate and accurate. To mitigate this issue, we contacted the respondents who gave ambiguous answers in the online questionnaire, but not all of them came back to us with a clearer reply. Lastly, it should be borne in mind that the 30 case studies selected represent only a fraction of currently active CS projects; this explains why statistically representative conclusions cannot be drawn from them.

## Comparative case study analysis: results and discussion

This section introduces the results of the data coding process and the findings of the comparative case study analysis, presented for each of the three research questions.

### Results of the data coding

The data collected from projects’ websites, secondary sources, and personal interviews were systematically organized in files set up for each CS project, and the resulting material was then manually coded in Microsoft Excel. More precisely, the data were first divided according to the three research questions—CSD purposes, SDG involvements, SDG opportunities and barriers—and subsequently classified into categories. To identify the categories, we relied on a data-driven inductive approach and combined ‘grounded or open coding’ (i.e. the analytical process by which codes are identified in the collected data) with ‘a priori coding’ (i.e. the analytical process by which codes are obtained from theory and existing literature) (Strauss and Corbin 1997; Ponti and Craglia 2020). This hybrid approach ensured that codes derived from the literature (see Table 2) were complemented by new codes emerging from the data. Once finalized, the codebook contained 18 categories (see Table S2 in the Supplementary Material). It should be noted that the data collected included statements that could be clustered into multiple categories. In other words, the data collected on the 30 CS projects amounted to more than 30 statements for each of the three research questions. In the case of CSD purposes, for instance, we have 67 relevant statements because some projects, such as FreshWater Watch, explicitly referred to more than one purpose for their CSD. For SDG involvements we have 32 statements; for SDG opportunities 35 statements; and for SDG barriers 34 statements. An overview of the main findings can be found in Table 5 and will be discussed in more detail in the following sections.

**Table 5:** Overview of the findings from the explorative case study analysis

Research question	Results
**CSD purposes** For which purposes do CS projects produce data?	Communities (28) Public governance (20) Scientific research (13) Statistics (6)
**SDG involvements** What types of involvements do CS projects have with the SDGs?	Superficial (19) Non-existent (8) Disappointing (3) Substantial (2)
**SDG opportunities and barriers** What do CS projects see as the main opportunities and barriers in linking CSD to the SDGs?	**Opportunities** Framework (12) Concrete support (8) Unsure (7) Non-existent (5) Data demand (3) **Barriers** Lack of knowledge (13) UN passivity (8) Political resistance (5) Technical (5) Non-existent (3)

### CSD purposes

The first empirical question in this explorative research asked: for which purposes do CS projects produce data? The analysis of the data reveals that CSD are produced for four main purposes: communities (28 cases), public governance (20 cases), scientific research (13 cases), and statistics (6 cases).

An overwhelming majority of initiatives justified the production of CSD using the positive impact of the data on communities. In this sense, CSD are primarily used to empower, engage, and educate citizens. In the words of the representative from *Africa’s Voices*: “We gather citizen data in places often difficult to reach by sustaining large-scale conversations over the radio and launching SMS open-ended surveys on crucial issues, such as the impact of COVID-19 on specific African regions. These data are meant to spur communities to participate in politics and put their voices at the heart of the continent’s transformation” (Interviewee IN05—see Table 4 for code explanation). The interviewee from* iNaturalist*, an app that collects people’s observations of plants and animals, stresses the educational value of the data: “We believe that recording information about nature has the tremendous potential to connect people to the environment and educate them about the importance of protecting it” (IN04).

The second most popular use of CSD is related to public governance. Several initiatives produce data in order to facilitate collaborative, accountable, and transparent governance and to raise public officials’ awareness of certain issues. One example comes from *Hush City*, an app through which individuals map and assess quiet areas in cities (i.e. areas with good acoustic and environmental qualities), thus generating data that can be integrated into city planning processes and inform policies for healthier living. Given its feasibility and replicability, *Hush City* has been adopted by the Berlin Senate in Germany for the preparation of the ‘Berlin Noise Action Plan 2019–2023’, and by the City Council of Limerick in Ireland for involving citizens in mapping and assessing quiet areas. Another example is *FixMyStreet*, a UK app that gathers citizen reports on street problems (such as broken streetlights or paving slabs) and sends them to local councils to improve the allocation of resources. These two representative examples illustrate a general pattern: most of the projects who mentioned public governance among their CSD purposes are primarily concerned with the impact of the data on local policymaking.

Besides community and public governance aims, some initiatives also seek to advance scientific research. In the case of *Stall Catchers*, for instance, individuals play an online game to identify stalls (obstructions) in blood vessels and considerably speed up Alzheimer’s research. The project *Did you feel it?* instead gathers reports from people who experience earthquakes around the world; the data, which consist of information on ground shaking and damages, are then used to enrich research in earthquake science.

Lastly, a few CS projects go beyond producing data for enhancing scientific progress and place noteworthy emphasis on the data themselves. In other words, these initiatives aim to utilize CSD to contribute to recognized datasets, actively fill data gaps in official statistics, and validate official data sources. Among them, two mentioned that their data are expressly intended to measure specific SDG indicators. The first one, *FreshWater Watch*, provides volunteer groups around the world with a simple test kit to measure water turbidity and nitrate pollution in local water streams. With almost 25,000 data points, “the CSD produced by *FreshWater Watch* can feed directly in SDG indicator 6.3.2”^13^ (IN07). The platform *LANDex* instead gives people the possibility to submit land data that monitor, for instance, tenure security, women’s land rights, and recognition of indigenous lands. It relies on citizen-led indicators whose data collection methodologies are similar to SDG methodologies. According to its founder, “the core mission of *LANDex* is to fill data gaps in land-related SDG indicators by collecting evidence from communities often absent from official statistics” (IN08).

In sum, CS projects primarily produce data to generate a positive impact on their communities, influence local public governance, and advance scientific progress—and a few are concerned with improving existing statistics. The following analyses directly turn to the CSD-SDG link.

### SDG involvements

The second question in this explorative research was: What types of involvements do CS projects have with the SDGs? We found that almost all have superficial (19 cases) or non-existent (8 cases) engagements with the SDGs; three further cases had been involved with the SDGs in the past but had revised their engagements as a result of disappointing experiences with some UN agencies. In congruence with the findings for the first research question, in only two cases (*LANDex* and *FreshWater Watch*) did respondents confirm having substantial involvements with the SDGs, since their initiatives produce data that feed directly into certain SDG indicators.

Although the majority of respondents answered positively to the question “Is your project familiar with the 17 SDGs?”, most of them claimed that their involvements with the SDGs are rather superficial. Within these initiatives, the SDGs are seldom mentioned and are used only implicitly. For instance, the founder of the platform *OpenLitterMap*, devoted to fighting plastic pollution, explains that “although our platform can potentially promote several SDGs, we never explicitly engage with them. The only instances in which the SDGs were relevant for us is when we applied for some EU grants that asked to specify the contribution of *OpenLitterMap* to the SDGs. I had to improvise my answers” (IN06). Interestingly, we found that six out of the seven CS case studies that explicitly refer to the SDGs on their websites have, nevertheless, only limited engagements with the UN goals (*LANDex* being the only exception). For instance, the representative from *Safecast*, an organization established after the Fukushima nuclear disaster in Japan to make up for the lack of trustworthy radiation data, stated, “When the SDGs were announced, some people started to ask about them and this is why we decided to include them on our website; but we haven’t put any particular thought into them or seen any change since then” (GF11). A similar experience is found in the case of *SciStarter*, a US-based platform connecting individuals to CS projects: “The SDGs are basically an after-thought. When a meeting is finished, we occasionally ask ourselves: did we do anything for the SDGs? And then we sometimes publish a blog post, but not much more than that” (IN01).

Three respondents also reported having had disappointing interactions with UN agencies in the past. Among them, *WikiMafia*, a free crowdsourced encyclopedia devoted to informing and mapping Mafia-related activities in Italy, was invited by the United Nations Office on Drugs and Crime (UNODC) to apply for the Education for Justice initiative, in the context of SDG 16^14^. One interviewee (IN02) laments: “Despite devoting hours and hours to the application, the UNODC never got back to me. I tried to contact them and ultimately assumed that they were never really interested in my project”. The representative from *ZanzaMapp*, a project that maps the location of mosquitoes to limit the spread of diseases, recalled his experience with the Global Mosquito Alert Consortium (GMAC), a platform dedicated to improving mosquito monitoring worldwide by bringing together the work of multiple organizations and sharing the data with the UN Environment Programme (UNEP): “Although it appears like there is one, the GMAC does not exist—it is only a façade. Only one workshop was organized by the UNEP in Geneva, but no concrete results were achieved and it became clear that the UN had no intention to really support and finance this initiative”. This casts doubts on the assertions of Fraisl et al. (2020, p. 10) and other scholars (Tyson et al. 2018), who claim that the GMAC could be purposely used to contribute to the SDG monitoring framework.

The findings of this empirical analysis reveal that only very few initiatives have substantial involvements with the SDGs, whereas the vast majority of them engage with the goals only superficially or do not engage at all. In addition, disappointing experiences with UN agencies can discourage those working with CSD from establishing tighter connections with the SDGs.

### SDG opportunities and barriers

The last empirical question in this explorative research was: What do CS projects see as the main opportunities and barriers in linking CSD to the SDGs? Among the opportunities, the SDGs can help CS projects by providing a legitimizing framework (12 cases), attracting concrete forms of support (8 cases), and increasing data demand (3 cases). Seven respondents were unsure whether the SDGs can provide any opportunity at all, while five identified no opportunity. Among the barriers, respondents cited lack of knowledge (13 cases), UN passivity (8 cases), political resistance (5 cases), and technical reasons (5 cases). Three respondents mentioned that they do not see any barriers.

There was widespread consensus that the SDGs can bring benefits to CS projects by providing a globally recognized framework. In this sense, the SDGs can set guidelines, incentivize communities, and confer legitimacy on CS initiatives. The idea of the SDGs as a legitimizing force is expressed, for instance, in the words of the representative from *QuestaGame*, a multiplier online game in which individuals compete to take pictures and identify rare plants and animals: “The SDGs give us a sense of legitimacy, ensuring our community that protecting biodiversity is not something we randomly came up with, but something the world came up with” (IN13).

Other respondents believe, instead, that a tighter link to the SDGs can also bring concrete forms of support—especially in terms of funding, visibility, and partnerships. For instance, the leader of the project *Nepal Monitor*, which gathers reports from Nepali citizens of any sort of violence-related incidents (such as gender-based violence or domestic violence), recalled that “when the UN Special Rapporteur on violence against women referenced the work of *Nepal Monitor* in the 2018 country analysis, our initiative gained international attention” (GF16).

Next to abstract incentives and concrete support, respondents from three organizations (*FreshWater Watch*, *LANDex*, and *Land Matrix*) acknowledged that the SDGs triggered an increase in data demand. “The SDGs have renewed the demand for good data that can inform action and measure progress towards their achievement. Without the SDGs, many data gaps would have never turned from invisible to visible”, explains the founder of *Land Matrix*, a platform that collects CSD on any type of large-scale land acquisitions (IN09). However, these three were outliers rather than representative cases. In fact, several respondents were completely unaware of the data-driven nature of the SDGs and bewildered by the idea that the goals are actually measurable.

Turning to the barriers, lack of knowledge of the SDGs was the most mentioned, as described by the respondent from *I Like Clean Air*, an initiative that aids parents and children in monitoring air pollution around schools in London: “To be honest, I think that only a very small percentage of organizations fully understand the SDGs and ordinary people are often unaware of their existence. Why should we use them if no one knows about them?” (GF15). This representative quote underlines the fact that not only are several projects unaware of the SDGs, but they also claim that their communities know little or nothing about them.

On top of lack of knowledge, a considerable group of participants reported feelings of frustration towards the UN. More precisely, they emphasized that the UN has so far only paid lip service to the idea of complementing official statistics with CSD, supporting CS projects in words but not giving any practical aid or laying out a roadmap with concrete steps towards increased collaboration. The statement of the respondent of *Map Kibera*, which uses inputs from citizens to create digital maps of the largest slum in Nairobi, captures this idea: “Since 2015, there has been a lot of talk within UN agencies about linking CSD to the SDGs, but we have seen no action. We assume that the UN networks are practicing business as usual and aren’t really serious about reaching out and engaging with true local organizations” (GF14).

Other respondents instead raised political concerns and explained that their communities or partners (including local governments) may oppose the SDGs and the UN. For instance, the spokesman from the Colorado State University project *CoCoRaHS*—which gathers over 50 million observations on rain, hail, or snow precipitations across North America—pointed out: “A good percentage of our volunteers hold conservative views and don’t want to hear anything about environmental issues or climate change. ‘Sustainable Development Goals’ would be suspicious to them” (GF13). But resistance to the SDGs does not only pertain to the political right. “If we would use our data to monitor the SDGs, I am sure that we would face considerable resistance from some industries and patient organizations—especially those from the far-left end of the political spectrum who are skeptical about international institutions such as the UN”, remarked the representative from *EUPATI*, which collects patients’ experiences with diseases and therapies with the aim of promoting people-centred medical research (GF08). For the representative of *Nepal Monitor*, the opposition to the SDGs comes from local governments themselves, which “might be bothered by the SDGs and do not always allow us to work openly with them, since local public officials do not have a positive impression of non-governmental organizations supported by foreign donors”.

Lastly, an exiguous number of representatives identified some technical barriers, mainly related to their organizations’ lack of resources or the non-standardized nature of their data. “We have thought about incorporating the SDGs, but our organization’s relatively small size and very limited capacity prevents us from integrating more of the SDGs”, emphasized the staff representing *WikiRumours*, a project that relies on citizens to gather instances of rumours and misinformation circulating both online and offline amongst a given community to prevent intercommunal conflicts (GF06).

By analysing the data collected on SDG opportunities, we found that CS projects value the SDGs because they can provide a global framework, some forms of concrete support, and fuel the demand for data. Among the SDG barriers, representatives mentioned widespread lack of knowledge, UN passivity, political resistance to the SDGs, and technical obstacles.

### Discussion of the comparative case study analysis

The comparative case study analysis provided insights into the CSD-SDG link from the perspective of CS projects. The findings empirically confirmed categories derived from the literature (Table 2) and identified new categories of explanation (Table 5).

In line with the literature reviewed, we found that CS projects produce data to positively impact communities (Kieslinger et al. 2017; Turrini et al. 2018), public governance (Meijer and Potjer 2018; Ponti and Craglia 2020), and scientific research (Newman et al. 2017; Turbé et al. 2019). We also identified a new category, which we called Statistics, in which we grouped all the initiatives that place significant relevance on the data themselves. On the basis of the literature and these findings, two general observations can be made that are of relevance for the CSD-SDG link: firstly, CS projects are largely interested in generating *local* impacts—whether on communities, governments, or both. This local focus can be purposefully exploited to improve the SDG monitoring framework, by compensating for the current lack of granular and spatially disaggregated data (MacFeely 2018; Yamin 2019), as well as helping to meet the promise of inclusive development (Fritz et al. 2019). Crucially, this entails that CS projects may not only ameliorate the current SDG data apparatus but also make significant, tangent contributions towards the broader idea of the SDGs. However, a second observation is that only a minority of initiatives currently produce data with the intention of filling gaps in existing datasets, and only a couple include the monitoring of SDG indicators among their primary aims. This suggests that there is still a significant lack of alignment between SDG data demand, on the one hand, and CSD purposes, on the other (Schade et al. 2020; See et al. 2020).

Turning directly to the CSD-SDG link, our analysis supports the idea that most CS projects have only superficial engagements with the SDGs—usually limited to grant applications and occasional website mentions. We also highlighted the disappointing interactions of a handful of initiatives with UN agencies, since these played a role in lowering their level of engagement with the SDGs. In general, these findings empirically underpin other researchers’ observations that the link between CSD and the SDGs is currently flimsy, with the UN goals occupying only a marginal position within citizen science (Lämmerhirt et al. 2017; Schade et al. 2020).

Delving deeper into the complexities of the CSD-SDG link, our findings lend empirical support to the grey literature which claims that CS projects recognize in the SDGs a promising opportunity to work within a global framework and to attract funding, attention, and partnerships (Lämmerhirt et al. 2017, 2018). Moreover, our analysis highlights that a couple of organizations value the SDGs because of the demand for data arising from them, but we also found that several respondents were unaware that the SDGs are actually measurable. Tracing back the foundations of this confusion, we identified a lack of knowledge of the SDGs as the most prominent barrier hindering CS projects from linking their work to the SDGs (Lämmerhirt et al. 2017). On top of that, we classified two new barriers: UN passivity, in which we grouped representatives who expressed frustration at the inaction of the UN; and political resistance, which included initiatives that were concerned with the political status of the UN. Put together, the findings from the last empirical question suggest that the SDGs could offer CS initiatives the opportunity to gain a global reach, both through abstract (framework) and more concrete means (funding, visibility, and partnerships); however, the barriers blocking these projects from engaging with the SDGs might be deep-rooted and hard to overcome.

## Conclusions

The SDGs aim to create people-centred development and were built on ambitious promises to “make all voices count” and “leave no one behind” (UNGA 2015). These promises are far from being met: the official data used to monitor SDG indicators do not cover remote locations, capture the issues of marginalized populations, or represent local communities. Hence, a growing body of scholarship is proposing to complement official data with citizen science data: data that citizens voluntarily generate to demand and drive change around issues important to them. The current article emerged from this call. The ultimate goal of this research was to develop a systematic empirical understanding of the link between CSD and the SDGs by listening to the voices of crucial stakeholders: CS projects.

We conducted an explorative comparative case study analysis to develop this understanding (*n* = 30). The empirical research confirmed expectations derived from the literature: CS projects have powerful local impacts since they aim to educate and engage communities and even shape local policymaking. For this reason, CSD can provide a much-needed local and contextual dimension to SDG global monitoring. At the same time, it has become very clear that the orientation of these projects is currently not towards the SDGs. Although some recognize that the SDGs provide a useful framework and concrete forms of support, many establish only feeble relations with the UN goals. Our research highlights that, in addition to technical reasons, this misalignment might be related to widespread lack of knowledge of the SDGs; political resistance towards the UN; and pervasive frustration arising from the UN’s inability to transform its ambitious, rhetorical promises into actions.

This article’s first contribution is empirical in nature: on the one hand, its findings empirically confirm the existence of some categories of CSD purposes, SDG involvements, and SDG opportunities and barriers that have hitherto been only theoretically or unsystematically studied. On the other hand, it identifies several new categories of explanation that have not yet been discussed in the literature. This range of identified explanations could provide fertile ground for researchers interested in the link between CSD and the SDGs, as well as in broader questions germane to the integration of citizen science within the context of SDG monitoring. In particular, this research could be used as a blueprint for developing quantitative analyses on the relationship between specific features of CS initiatives (e.g. organization category, size, geographic coverage) and particular categories of SDG involvements, SDG opportunities, and SDG barriers. For instance, an interesting avenue for further research might involve investigating whether categories such as Framework or Concrete Support are influenced by the type or size of organization.

Another contribution of this article is that it sharpens our theoretical understanding of CSD as positioned within the SDG monitoring framework (Fraisl et al. 2020; Fritz et al. 2019). In particular, it stresses the local orientation and highlights the embeddedness of CS projects in their communities, thus touching on the potential of CSD to surface counternarratives of development and to correct power asymmetries resulting from an overreliance on official statistics (Fisher and Fukuda-Parr 2019; Yamin 2019). In this sense, it broadens the scope of CSD and encourages other researchers to study CSD within the paradigm of the SDGs.

This article’s third contribution lies in its novel approach. Instead of examining the CSD-SDG link from the perspective of the SDGs, it takes a bottom-up approach and gives voice to the positions of CS projects on the SDGs—positions so far largely neglected. Through this approach, this article shows that the barriers separating CS initiatives from the SDGs do not only come from the ‘SDG side’ (including, for instance, issues related to quality control for CSD or the standardization of data collection methodologies) (see Cázarez-Grageda et al 2020; Fritz et al. 2019; Fraisl et al. 2020). In fact, even if the ‘SDG barriers’ could be gradually overcome, CS projects might still refrain from linking their data to the SDGs because, for instance, they are unfamiliar with the goals, not interested in them, or even unwilling to contribute. This is why it is paramount that UN policy makers, and in particular the civil servants involved in managing national statistics, put themselves more in ‘the heads of CS projects’ and acknowledge that the link between CSD and the SDGs cannot be established unless these initiatives are also engaged and on board (e.g. through collaborative data working groups^15^).

To better engage CS projects, a useful first step involves shifting the framing commonly used in research and policy circles to describe the CSD-SDG relationship from ‘the SDGs can benefit from citizen science’ (Fraisl et al. 2020) to ‘the SDGs and citizen science can benefit from each other’. Stressing the mutually beneficial nature of the CSD-SDG link could help the UN, governments, and experts, on the one hand, and CS initiatives and their communities, on the other, to move closer and faster towards each other.^16^ A second, necessary step would be to meticulously unpack the barriers that CS projects face in relation to the SDGs and understand their nature (data-related, practical, political, legal, knowledge-related, etc.). The innovative approach taken in this article reveals that deep-seated obstacles seem to exist; however, the limitations in terms of methodology and scope do not allow for a nuanced understanding of these barriers, leaving open questions about their representativeness and relative weight. Further bottom-up, qualitative research is needed, which can enhance our understanding of these barriers and ultimately provide recommendations on how to best tackle them.

In sum, what we have attempted to show in this article is that listening to and engaging with CS projects can add a multifaceted, indispensable layer of understanding to the link between citizen science data and the Sustainable Development Goals. On the long and winding road of sustainable and inclusive development, CS initiatives can provide an invaluable contribution to SDG monitoring. But they can do so only if we truly make their voices count.

## Supplementary Information

Below is the link to the electronic supplementary material.

Supplementary file1 (DOCX 14 kb)

## Footnotes

1 In the report *A World That Counts*, NSOs are presented as the “guardians of public data for the public good” and “central to the whole of government efforts to harness the data revolution for sustainable development” (IEAG 2014, p. 9). Satterthwaite and Dhital therefore refer to the NSOs as the ultimate “arbiters of data for monitoring the SDGs” (2019, p. 101).

2 For instance, MacFeely (2019) shows that as of 2018, data for only 40 percent of the SDG indicators could be compiled. Similarly, the report by the Danish Institute for Human Rights (DIHR 2017) cites the case of Denmark—one of the countries with the largest statistical capacity—where the NSO has readily available data for only 77 of the 232 indicators, and the case of Bangladesh where no data is available for 121 of the 169 targets.

3 The literature does not yet provide a comprehensive and widely accepted definition of CSD and the term ‘citizen science data’ (which we adopted following Bishop et al. 2020) is often used interchangeably with the following terms: citizen-generated data (Lämmerhirt et al. 2017, 2018), citizen-gathered data (Gabrys et al. 2016), citizen-generated open data (Meijer and Potjer 2018), citizen-science generated data (Jordan et al. 2015), citizen data (Purdam 2014), community-generated data or community-based data (Carlson and Cohen 2018; Winkler and Satterthwaite 2017).

4 For detailed definitions and discussion of big data see Kennedy et al. (2015), Kitchin and McArdle (2016), MacFeely (2019), and Michael and Lupton (2016). For an interesting example of the use of big data in the context of global health metrics, see Mahajan (2019).

5 Despite the fact that projects that rely on ‘citizen scientists’ and use some sort of CSD have existed for decades, the term ‘citizen science’ has emerged only recently and researchers are still struggling to establish citizen science as a distinct scientific field of inquiry (Kosmala et al. 2016; Strasser et al. 2019).

6 See https://ebird.org/home.

7 See https://indigenousnavigator.org.

8 See https://www.coronareport.global.

9 In 2018, the European Commission Joint Research Centre (JRC) published the first inventory of *environmental* CS projects (see European Commission 2018a); however, no database currently exists that includes projects from multiple sectors (Ponti and Craglia 2020).

10 These keywords were chosen following Meijer and Potjer (2018) and Ponti and Craglia (2020).

11 The organization attributes (e.g. organization category) and the attributes’ characteristics (e.g. governmental, academic, consortium, etc.) were adapted from: ‘Inventory of environmental citizen science projects – Attributes for the inventory developed and published by the JRC’ (European Commission 2018b); Meijer and Potjer (2018); and Ponti and Craglia (2020).

12 See, for instance, Kling and Pozdnoukhov (2012) and Li et al. (2016), who use the term CSD but mainly refer to cases of ‘big data’ (Gray et al. 2016).

13 SDG 6.3.2: “Portion of bodies of water with good ambient water quality” (UNGA 2015).

14 SDG 16: "Promote peaceful and inclusive societies for sustainable development, provide access to justice for all and build effective, accountable and inclusive institutions at all levels” (UNGA 2015).

15 We are grateful to one of the anonymous reviewers for this suggestion.

16 The recently launched World Development Report 2021 (World Bank 2021, pp. 313-315) provides good starting points on how to integrate civil society in the national data system.

## References

[CR1] Bett K, Wood C, Fraisl D (2020) WeObserve SDGs CoP. May 2020 Meeting [Webinar], WeObserve. https://docs.google.com/document/d/1RiWb1kF7EJ1c-eRMyhxGbreKOVPT9yx_kiXuLaJeXSo/edit

[CR2] Biernacki P, Waldorf D (1981). Snowball sampling: problems and techniques of chain referral sampling. Sociol Methods Res.

[CR3] Bio Innovation Service (2018) Citizen Science for Environmental Policy: Development of an EU-wide Inventory and Analysis of Selected Practices. Final Report for the European Commission, DG Environment under the contract 070203/2017/768879/ETU/ ENV.A.3, in collaboration with Fundacion Ibercivis and the National History Museum

[CR4] Bishop IJ, Warner S, van Noordwijk TCGE, Nyoni FC, Loiselle S (2020). Citizen science monitoring for sustainable development indicator 6.3.2 in England and Zambia. Sustainability.

[CR5] Blaney RP, Pocock M, Jones GD (2016) Citizen science and environmental monitoring: towards a methodology for evaluating opportunities, costs and benefits. http://www.ukeof.org.uk/resources/citizen-science-resources/Costbenefitcitizenscience.pdf

[CR6] Bonney R, Cooper CB, Dickinson J, Kelling S, Phillips T, Rosenberg KV, Shirk J (2009). Citizen science: a developing tool for expanding science knowledge and scientific literacy. Bioscience.

[CR7] Bonney R, Cooper C, Ballard H (2016). The theory and practice of citizen science: launching a new journal. Citiz Sci Theory Pract.

[CR8] Burgess HK, DeBey LB, Froehlich HE, Schmidt N, Theobald EJ, Ettinger AK, HilleRisLambers J, Tweksbury J, Parrish JK (2017). The science of citizen science: exploring barriers to use as a primary research tool. Biol Cons.

[CR9] Carlson T, Cohen A (2018). Linking community-based monitoring to water policy: perceptions of citizen scientists. J Environ Manag.

[CR10] Cázarez-Grageda K, Schmidt J, and Ranjan, R (2020). Reusing Citizen-Generated Data for Official Reporting: A Quality Framework for National Statistical Office–Civil Society Organization Engagement’, PARIS21 Working Paper, PARIS21 Consortium, Paris. Available at: https://paris21.org/sites/default/files/inline-files/CGD_FINAL.pdf

[CR11] Cohn JP (2008). Citizen science: can volunteers do real research?. Bioscience.

[CR12] Daguitan F, Lehohla P, Mwangi C et al. (2019a) The current state of our data and knowledge. In: Global environment outlook healthy people. UN Environment, Nairobi

[CR13] Daguitan F, Mwangi C, Tan MG et al. (2019b) Future data and knowledge needs. In: Global environment outlook—GEO-6: healthy planet, healthy people. UN Environment, Nairobi

[CR14] Danielsen F, Burgess ND, Balmford A (2005). Monitoring matters: examining the potential of locally-based approaches. Biodivers Conserv.

[CR15] Danish Institute for Human Rights (DIHR) (2017) Human rights and data: tolls and resources for sustainable development. http://sdghelpdesk.unescap.org/e-library/human-rights-and-data-tools-and-resourcessustainable-development-2017

[CR16] DataShift (2017) Using citizen-generated data to monitor the SDGs. A tool for the GPSDD data revolution roadmaps toolkit. https://www.data4sdgs.org/sites/default/files/2017-09/Making%20Use%20of%20Citizen-Generated%20Data%20-%20Data4SDGs%20Toolbox%20Module.pdf

[CR17] Eisenhardt KM (1989). Building theories from case study research. Acad Manag Rev.

[CR18] Eitzel MV, Cappadonna JL, Santos-Lang C (2017). Citizen science terminology matters: exploring key terms. Citiz Sci Theory Pract.

[CR19] European Commission (2018a) An inventory of citizen science activities for environmental policies. https://data.jrc.ec.europa.eu/dataset/jrc-citsci-10004

[CR20] European Commission (2018b) An inventory of environmental citizen science projects—attributes for the inventory developed and published by the JRC. https://data.jrc.ec.europa.eu/dataset/jrc-citsci-10004

[CR21] Fisher A, Fukuda-Parr S (2019). Introduction—data, knowledge, politics and localizing the SDGs. J Hum Dev Capab.

[CR22] Flückiger Y, Seth N (2016). SDG indicators need crowdsourcing. Nature.

[CR23] Fraisl D, Campbell J, See L (2020). Mapping citizen science contributions to the UN sustainable development goals. Sustain Sci.

[CR24] Freitag A, Meyer R, Whiteman L (2016). Strategies employed by citizen science programs to increase the credibility of their data. Citiz Sci Theory Pract.

[CR25] Fritz S, See L, Carlson T (2019). Citizen science and the united nations sustainable development goals. Nat Sustain.

[CR26] Fukuda-Parr S, McNeill D (2019). Knowledge and politics in setting and measuring the SDGs: introduction to special issue. Glob Policy.

[CR27] Gabrys J, Pritchard H, Barratt B (2016). Just good enough data: figuring data citizenships through air pollution sensing and data stories. Big Data Soc.

[CR28] Gray J, Lämmerhirt D, Bounegru L (2016) Changing what counts. How can citizen-generated and civil society data be used as an advocacy tool to change official data collection. https://papers.ssrn.com/sol3/papers.cfm?abstract_id=2742871

[CR29] Haklay M (2020) Introducing: ECSA Characteristics of Citizen Science. https://povesham.wordpress.com/2020/05/04/introducing-ecsa-characteristics-of-citizen-science/

[CR30] Independent Expert Advisory Group on a Data Revolution for Sustainable Development (IEAG) (2014) A world that counts: mobilising the data revolution for sustainable development. https://www.undatarevolution.org/wpcontent/uploads/2014/11/A-World-That-Counts.pdf

[CR31] Jordan R, Crall A, Gray S, Phillips T, Mellor D (2015). Citizen science as a distinct field of inquiry. Bioscience.

[CR32] Kennedy H, Poell T, van Dijck J (2015). Data and agency. Big Data Soc.

[CR33] Kieslinger B, Schaefer T, Heigl F, Dörler D, Richter A, Bonn A (2017) The challenge of evaluation: an open framework for evaluating citizen science activities. https://osf.io/preprints/socarxiv/enzc9

[CR34] Kitchin R, McArdle G (2016). What makes big data, big data? Exploring the ontological characteristics of 26 datasets. Big Data Soc.

[CR35] Kling F, Pozdnoukhov A (2012) When a city tells a story: urban topic analysis. In: Proceedings of the ACM International Symposium on Advances in Geographic Information Systems. 10.1145/2424321.2424395

[CR36] Kosmala M, Wiggins A, Swanson A, Simmons B (2016). Assessing data quality in citizen science. Front Ecol Environ.

[CR37] Lämmerhirt D, Jameson S, Prasetyo E (2017) Acting locally, monitoring globally? Report for DataShift. https://civicus.org/thedatashift/wp-content/uploads/2017/03/Acting-locally-monitoring-globally_Full-Report.pdf

[CR38] Lämmerhirt D, Gray J, Venturini T, Meunier A (2018) Advancing sustainability together? Citizen-generated data and the sustainable development goals. Report for the global partnership for sustainable development data. https://www.data4sdgs.org/resources/advancing-sustainability-together-citizen-generated-data-and-sustainable-development

[CR39] Langford M, Winkler I (2014). Muddying the water? Assessing target-based approaches in development cooperation for water and sanitation. J Hum Dev Capab.

[CR40] Li Z, Wang C, Emrich CT, Guo D (2016). A novel approach to leveraging social media for rapid flood mapping: a case study of the 2015 South Carolina floods. Cartogr Geogr Inf Sci.

[CR41] Lowry CS, Fienen MN (2013). CrowdHydrology: crowdsourcing hydrologic data and engaging citizen scientists. Ground Water.

[CR42] Lukyanenko R, Parsons J, Wiersma Y (2011) Citizen science 2.0: data management principles to harness the power of the crowd. In: Jain H, Sinha AP, Vitharana P (eds) Service-oriented perspectives in design science research. DESRIST 2011. Lecture Notes in Computer Science 6629. Springer, Berlin Heidelberg, pp 465–473

[CR43] Lukyanenko R, Parsons J, Wiersma Y (2016). Emerging problems of data quality in citizen science. Conserv Biol.

[CR44] MacFeely S (2018) The 2030 agenda: an unprecedented statistical challenge. Paper for Friedrich Ebert Stiftung, Berlin. http://library.fes.de/pdf-files/iez/14796.pdf

[CR45] MacFeely S, Nastav B (2019). “You say you want a [data] revolution”: a proposal to use unofficial statistics for the SDG global indicator framework. Stat J IOS.

[CR46] Mahajan M (2019). The IHME in the shifting landscape of global health metrics. Global Pol.

[CR47] Meijer A, Potjer S (2018). Citizen-generated open data: an explorative analysis of 35 cases. Gov Inf Q.

[CR48] Merry SE (2019). The sustainable development goals confront the infrastructure of measurement. Global Pol.

[CR49] Michael M, Lupton D (2016). Toward a manifesto for the public understanding of big data. Public Underst Sci.

[CR50] Newman G, Chandler M, Clyde M, McGreavy B, Haklay M, Ballard H, Gray S, Scarpino R, Hauptfeld R, Mellor D, Gallo J (2017). Leveraging the power of place in citizen science for effective conservation decision making. Biol Cons.

[CR51] Ponti M, Craglia M (2020) Citizen-generated data for public policy. European Commission, ISPRA, 2020 JRC120231. https://ec.europa.eu/jrc/communities/en/community/digitranscope/document/citizen-generated-data-public-policy

[CR52] Purdam K (2014). Citizen social science and citizen data? Methodological and ethical challenges for social research. Curr Sociol.

[CR53] Rogers R (2013). Digital methods.

[CR54] Saner R, Yiu L, Nguyen M (2019). Monitoring the SDGs: digital and social technologies to ensure citizen participation, inclusiveness and transparency. Dev Pol Rev.

[CR55] Satterthwaite ML, Dhital S (2019). Measuring access to justice: transformation and technicality in SDG 16.3. Global Pol.

[CR56] Schade S, Lämmerhirt D, Fraisl D (2020) WeObserve SDGs CoP. May 2020 Meeting [Webinar], WeObserve. https://docs.google.com/document/d/1RiWb1kF7EJ1c-eRMyhxGbreKOVPT9yx_kiXuLaJeXSo/edit

[CR57] See L, Fraisl D, Holm J, de Sherbinin A, Putnum R (2020) Citizen science, community engagement and the United Nations Sustainable Development Goals [Webinar], Citizen Science Association. https://www.youtube.com/watch?v=zc6ir6E17gc&feature=youtu.be.

[CR58] Shirk JL, Ballard HL, Wilderman CC (2012). Public participation in scientific research: a framework for deliberate design. Ecol Soc.

[CR59] Silverton J (2009). A new dawn for citizen science. Trends Ecol Evol.

[CR60] Strasser BJ, Mahr D, Sanchez GA, Tancoigne E (2019). “Citizen Science”? Rethinking Science and Public Participation. Sci Technol Stud.

[CR61] Strauss A, Corbin J (1997). Grounded theory in practice.

[CR62] Sullivan BL, Wood C, Liff MJ (2009). eBird: a citizen-based bird observation network in the biological sciences. Biol Cons.

[CR63] Turbé A, Barba J, Pelacho M (2019). Understanding the citizen science landscape for European Environmental Policy: an assessment and recommendations. Citiz Sci Theory Pract.

[CR64] Turrini T, Dorler D, Richter A, Heigl F, Bonn A (2018). The threefold potential of environmental citizen science—generating knowledge, creating learning opportunities and enabling civic participation. Biol Conserv.

[CR65] Tyson E, Bowser A, Palmer J (2018). Global mosquito alert: building citizen science capacity for surveillance and control of disease-vector mosquitoes.

[CR66] UNGA (2015) A/RES/70/1 UN General Assembly Transforming our World: the 2030 Agenda for Sustainable Development. Seventieth session of the General Assembly on 25 September 2015

[CR67] Ward A, Hershaw E, Joshi DR, Odonchimeg O (2020), Leaning LANDex: generating people-centred data and report on SDG [Webinar]. https://www.youtube.com/watch?v=BuTrVxasXN0

[CR68] West S, Pateman R (2017) How could citizen science support the Sustainable Development Goals? Discussion brief for Stockholm Environment Institute. https://www.sei.org/mediamanager/documents/Publications/SEI-2017-PB-citizen-science-sdgs.pdf

[CR69] Wiggins A, Crowston K (2011) From conservation to crowdsourcing: a typology of citizen science. Paper presented at 44th Hawaii International Conference on System Sciences, Kauai. https://ieeexplore.ieee.org/document/5718708

[CR70] Winkler IT, Satterthwaite ML (2017). Leaving no one behind? Persistent inequalities in the SDGs. Int J Hum Rights.

[CR71] World Bank (2021) Data for Better Lives. World Development Report 2021. The World Bank, Washington

[CR72] Yamin AE (2019). Power, politics and knowledge claims: sexual and reproductive health and rights in the SDG era. Global Pol.

[CR73] Yap ML-M, Watene K (2019). The sustainable development goals (SDGs) and indigenous peoples: another missed opportunity?. J Hum Dev Capab.

[CR74] Yin R (2014). Case study research: design and methods.

