# Supplementary material for: Using citizen science data to monitor the Sustainable Development Goals: a bottom-up analysis
Source: Sustain Sci. 2021 Jul 23;16(6):1945–62. doi: 10.1007/s11625-021-01001-1 (PMC8298195; doi:10.1007/s11625-021-01001-1)
Supplement: Supplementary file 1 — Supplementary file1 (DOCX 14 kb) [file 11625_2021_1001_MOESM1_ESM.docx]

1. **Table S1.** Inventory of CS projects https://drive.google.com/file/d/1F2luYVBPxDudY9ZpWEuRbjjub9Ib91VK/view?usp=sharing
2. **Table S2.** Codebook

https://drive.google.com/file/d/1sHyCCWVlQp5NTc5_p4hpRGloox35fOf0/view
